# A new wireless ultrasound probe for ultrasound guided central venous access

**DOI:** 10.1186/2036-7902-6-S2-A12

**Published:** 2014-08-27

**Authors:** M Pittiruti, A Emoli, S Cappuccio, A La Greca

**Affiliations:** 1 Emergency Surgery Unit, “A. Gemelli” Hospital – Catholic University – Roma, Italy; 2 Dept. of Oncology, “A. Gemelli” Hospital – Catholic University – Roma, Italy

## Background

Ultrasound (US) is considered mandatory for placement of central venous access devices (VAD), both for venepuncture and for early detection of potential complications.

## Objective

We report our preliminary experience with a new US device equipped with a wireless linear transducer (Acuson Freestyle, Siemens), specifically made for US-guided procedures.

## Patients and methods

The US system we tested consists of a 5-13 MHz linear probe connected by wireless technology (8 GHz ultra wide band) to a compact, keyboardless device. The probe can be completely wrapped up in a sterile cover so to perform the procedure with maximal barrier precautions. The main functions (gain, depth, freeze, save, etc.) can be operated either by controls built-in in the transducer or directly on the screen of the device. We adopted this wireless systems in 38 consecutive VAD placements (25 PICCs, 12 ports and 1 cuffed-tunneled catheter). US was used for US-guided puncture and cannulation of different veins (20 basilic veins, 5 brachial veins, 9 axillary-subclavian veins, 3 internal jugular veins and 1 brachio-cephalic vein), for US detection of potential misplacement of the guidewire and/or of the catheter, as well as for ruling out pneumothorax by US scan of the pleura in the intercostal space.

## Results

All venepunctures were performed successfully. The image quality was excellent and there was no delay in the data trasmission to the screen. The transducer was easy to handle and to use. Visualization of needles, guidewires and catheters was optimal. All operators reported an increased easiness in performing the US-guided maneuvers, due to the absence of the cable.

## Conclusion

Our experience suggests that wireless US might be specifically useful in US-guided procedures. Some of its potential advantages are: maximal flexibility in placing the device and the probe, as their connection is wireless; complete freedom of movement for the operator during the procedure; optimal compliance with the current recommendations for infection control.

